# Enhanced low-temperature NH_3_-SCR performance of Ce/TiO_2_ modified by Ho catalyst

**DOI:** 10.1098/rsos.182120

**Published:** 2019-03-13

**Authors:** Ting-ting Zhang, Li-min Yan

**Affiliations:** Shanghai University Microelectronic R&D Center, Shanghai University, Shanghai 200072, People's Republic of China

**Keywords:** Ce/TiO_2_, Ho doping, SO_2_ resistance, low-temperature NH_3_-SCR

## Abstract

Holmium was used as a dopant to boost the low-temperature NH_3_-selective catalytic reduction (SCR) performance of Ce/TiO_2_ catalyst. It was ascertained that certain amount of Ho-doping species could exceedingly improve the low-temperature SCR activity under 60 000 h^−1^ of Ce/TiO_2_, accompanied with the improvement of tolerance to H_2_O and SO_2_ at 200°C. Characterization results manifested that Ho modification could not only result in inhibiting the growth of TiO_2_ crystals and the enlargement of specific surface area but also lead to the enhanced redox ability and the increased amount of surface-adsorbed substances, all of which could promote the low-temperature NH_3_-SCR performance of Ce/TiO_2_.

## 1. Introduction

Lately, NOx has become one of the significant sources of air pollution. The over-standard concentration of NOx emission was mainly caused by the combustion process of fossil fuel, which has caused many environmental problems such as city smog and pollution [1–4]. Selective catalytic reduction (SCR) is the widely accepted de-NOx technology, and V_2_O_5_-WO_3_ (MoO_3_)/TiO_2_ catalyst is the most commercially used catalyst in this SCR system [5]. However, there are still some disadvantages in the SCR system with V_2_O_5_-WO_3_ (MoO_3_)/TiO_2_, such as the high operating temperature (300–400°C), the toxicity of vanadium species, low N_2_ selectivity in the working temperature range [6–9]. Based on these practical disadvantages, it is necessary to study non-vanadium catalysts with better low-temperature SCR performance.

Cerium, one of the most abundant rare-earth metals, has drawn attention due to the high oxygen storage capacity and good redox property. It has been widely applied in catalysis, such as carbon monoxide oxidation and reforming reactions [10–12]. Results of previous research proved that cerium-based oxide catalysts had a good SCR performance. Gao *et al*. [13] reported that Ce/TiO_2_ by the sol–gel method possesses high surface area and good redox ability, contributing to its high SCR activity. Vuong *et al*. [14] reported that V/CeTiO_2_ catalysts showed excellent de-NOx activity at low temperature. Notably, the best one of these V/CeTiO_2_ catalysts showed almost 100% NO conversion at 190°C. It was also found [15] that doping certain quantity of Ca would increase Ce^3+^ and surface-adsorbed oxygen. Meanwhile, the Brønsted acidity and redox ability were also greatly enhanced. All these factors may be responsible for the enhanced activity. Mosrati [16] recently reported that an impregnated Ce/Ti oxide catalyst with Nb modification presents a 95% NOx conversion at 200°C. Relevant characterization results proved that the Nb introduction decreases the surface area and strengthens the surface acidity. A Ce–Ti oxide catalyst with Cu addition could promote the SO_2_ resistance of Ce–Ti oxide [17]. Although several catalysts, such as V/CeTiO_2_ and Ce–Cu–TiO_2_, have been successfully applied in NH_3_-SCR, enhanced low-temperature NH_3_-SCR performance and SO_2_ resistance of Ce/TiO_2_ modified by Ho have never been reported.

As a rare earth metal, Ho has been successfully applied for improving the photocatalytic activity of TiO_2_ [18]. Owing to its electron trap effect of Ho^2+^↔Ho^3+^, the doping of Ho could efficiently enhance the photocatalytic ability of TiO_2_. Gamal *et al*. [19] reported that the surface of Ho_2_O_3_ exposes more Lewis acid sites, which play a vital role in NH_3_-SCR reaction. It was also reported [20] that Ho-modified Fe–Mn/TiO_2_ catalyst shows a larger specific area of Fe_2_O_3_ phase compared with that of Fe–Mn/TiO_2_, which results in a board temperature window and high SO_2_ tolerance in NH_3_-SCR reaction. However, the investigation of Ce/TiO_2_ catalyst with Ho addition has not been reported. In this work, Ho is used for improving the low-temperature NH_3_-SCR activity of Ce/TiO_2_, and several characterization methods were applied for investigating the promotion mechanism. Furthermore, SO_2_ + H_2_O tolerance of the best catalyst was also studied.

## 2. Experimental

### 2.1. Catalyst preparation

The impregnation method was used to prepare the catalysts. Titanium dioxide (anatase, 0.05 mol) was impregnated with cerium nitrate (0.0175 mol) and holmium nitrate in 100 ml deionized water, followed by stirring at 20°C for 3 h. The obtained mixture was dried for 12 h at 100°C and then calcined at 500°C for 4 h. The prepared samples were labelled as Ce_0.35_/TiO_2_ and Ho_x_Ce_0.35_/TiO_2_ (the molar ratios of Ho/Ti and Ce/Ti were *x* and 0.35, respectively).

### 2.2. Catalyst characterization

Powder X-ray diffraction (XRD) patterns were obtained on a Philips X'pert Pro diffractometer with Ni-filtered Cu K*α* radiation (0.15408 nm). 2*θ* ranged from 10° to 80° with a step size of 0.02°.

The specific surface area was measured by N_2_ adsorption at −196°C, using an ASAP 2020 volumetric adsorption analyser. Before each precise test, the catalysts were evacuated for 3 h at 300°C. The specific surface area and the pore volume of the samples were calculated by the Brunauer–Emmett–Teller (BET) method and the pore size distributions were derived from the adsorption branches of the isotherms using the Barrett–Joyner–Halenda model.

The H_2_ temperature-programmed reduction (TPR) experiments were performed on a Micromeritics AutoChem 2920 chemisorption analyser. Typically, 0.1 g sample was pretreated in pure N_2_ at 400°C for 0.5 h and then cooled to 20°C followed by N_2_ purging for 0.5 h. The temperature was heated by 10°C min^−1^ from 100 to 800°C in 10 vol% H_2_/Ar. Thermal conductivity detector monitored H_2_ consumption in this progress.

The NH_3_ temperature-programmed desorption (TPD) experiments were carried out on the same equipment as the TPR experiment. As a pretreatment step, 0.1 g samples were purged at 400°C in N_2_ for 0.5 h and cooled to 30°C. Then the samples were purged in NH_3_ for 1.0 h. At last, the programmed desorption was carried out at the rate of 10°C min^−1^ (100–500°C) in Ar.

*In situ* diffuse reflectance infrared Fourier transform spectroscopy (DRIFTS) experiments were carried out on a Nicolet 6700 FTIR spectrometer with an MCT/A detector. As a pretreatment step, the catalysts were treated at 450°C in N_2_ for 0.5 h and cooled to 50°C. Background spectra were recorded in the N_2_ flow and automatically subtracted from the corresponding spectra. The spectra were recorded by accumulating 64 scans at a 4 cm^−1^ resolution.

### 2.3. Catalytic activity test

SCR activity experiments were performed in a fixed-bed reactor containing 0.4 g catalysts (40–60 mesh) with a GHSV of 60 000 h^−1^. The total gas flow was 200 ml min^−1^, which was premixed in a gas mixer to obtain the simulated gas of [NO] = [NH_3_] = 500 ppm, [O_2_] = 3 vol.%, [H_2_O] = 8 vol.% (when used), [SO_2_] = 200 ppm (when used) and balanced by N_2_. Then the mixed gas went into the reactor and the NO and NO_2_ concentrations were monitored by a 350-XL flue gas analyser. The experiment data were recorded from 100 to 400°C at a steady state. The formulae for NO_x_ conversion and N_2_ selectivity were as follows:

2.1 $${\mathrm{NO}}_{x}\;\mathrm{conversion}(\text{\%})=\frac{{\left[{\mathrm{NO}}_{x}\right]}_{\mathrm{in}}-{\left[{\mathrm{NO}}_{x}\right]}_{\mathrm{out}}}{{\left[{\mathrm{NO}}_{x}\right]}_{\mathrm{in}}}\times 100\text{\%}$$

2.2 $$N_{2}\mathrm{selectivity}=\left(1-\frac{2{\left[N_{2}O\right]}_{\mathrm{out}}}{{\left[\mathrm{NOx}\right]}_{\mathrm{in}}+{\left[NH_{3}\right]}_{\mathrm{in}}-{\left[\mathrm{NOx}\right]}_{\mathrm{out}}-{\left[NH_{3}\right]}_{\mathrm{out}}}\right)\times 100\text{\%}.$$

Also, NO oxidation conversion was also tested in the same fixed-bed reactor in the same simulated flue gas components without NH_3_.

## 3. Results and discussion

### 3.1. Catalytic performance

**Figure 1.**
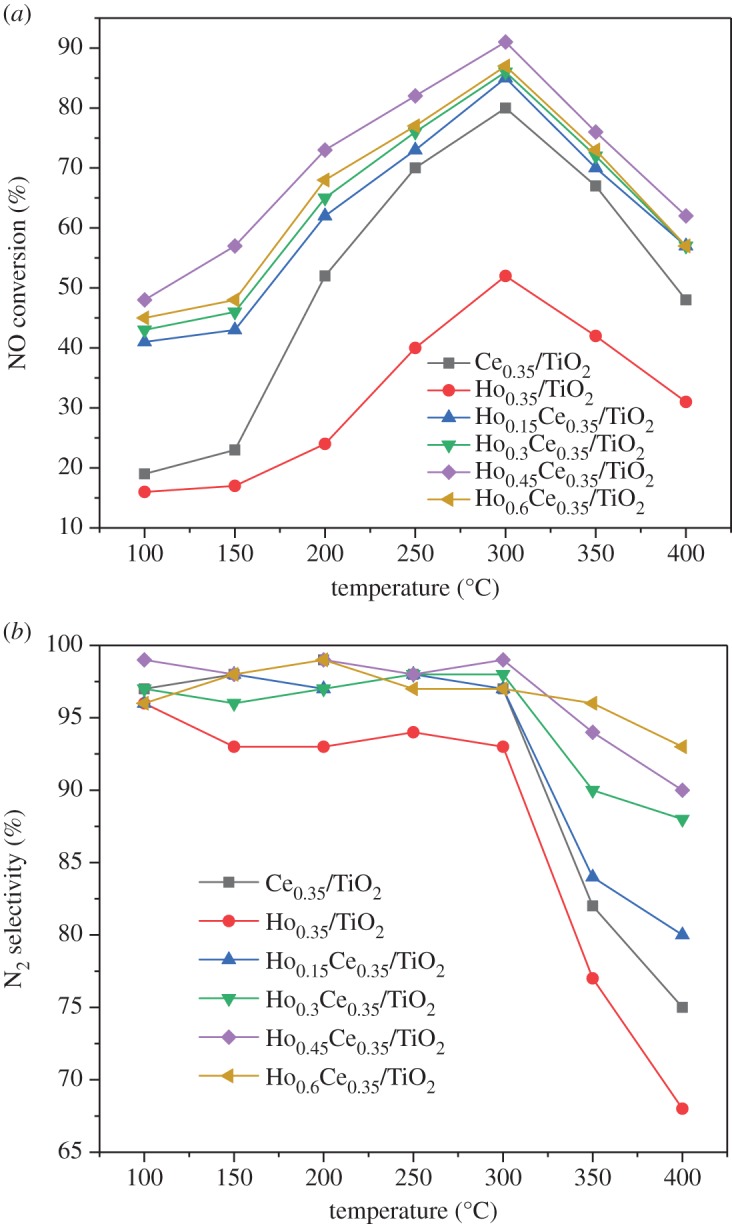
(*a*) NOx conversion and (*b*) N_2_ selectivity in the NH_3_-SCR reaction over Ho_x_Ce_0.35_/TiO_2_ catalysts (500 ppm NO, 500 ppm NH_3_, 3 vol.% O_2_, total flow rate 200 ml min^−1^ and GHSV = 60 000 h^−1^).

The NOx conversions of various catalysts are plotted as a function of temperature, as exhibited in figure 1*a*. Among the prepared catalysts, Ce_0.35_/TiO_2_ and Ho_0.35_/TiO_2_ showed a limited de-NOx activity (less than 80%) in the entire temperature scope. It is notable that the low-temperature (less than 200°C) catalytic activity of Ce_0.35_/TiO_2_ was much improved when small amounts of Ho species are doped, as evidenced by the NO conversion of Ho_0.15_Ce_0.35_/TiO_2_. When the Ho/Ti molar ratio rises to 0.45, the NOx conversion over Ce_0.35_/TiO_2_ at 150°C was also increased from 22% to 56%. However, further increasing of Ho/Ti molar ratio to 0.6 led to a slight decrease of de-NOx activity in the whole temperature range. Figure 1*b* shows the N_2_ selectivity as a function of temperature over Ho_x_Ce_0.35_/TiO_2_ catalysts. It could be readily observed that the addition of Ho could enhance the N_2_ selectivity of Ce_0.35_/TiO_2_ catalyst. Although all prepared catalysts showed high N_2_ selectivity in the temperature range of 100–300°C, Ce_0.35_/TiO_2_ added with Ho exhibited relatively better N_2_ selectivity above 300°C compared with Ce_0.35_/TiO_2_ catalyst.

### 3.2. Tolerance of SO_2_ and H_2_O

**Figure 2.**
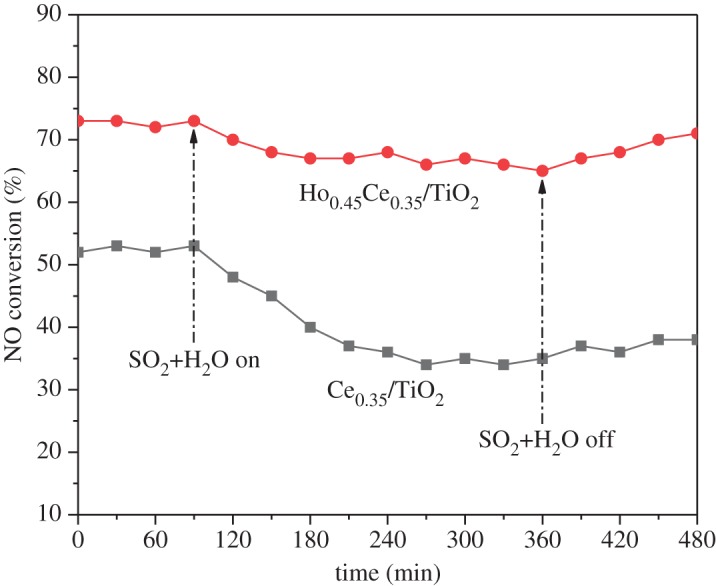
NOx conversion of Ce_0.35_/TiO_2_ and Ho_0.45_Ce_0.35_/TiO_2_ in the presence of SO_2_ and H_2_O at 200°C (500 ppm NO, 500 ppm NH_3_, 3 vol.% O_2_, 8 vol.% H_2_O, 200 ppm SO_2_, total flow rate 200 ml min^−1^ and GHSV = 60 000 h^−1^).

In practical applications, trace amounts of sulfur dioxide and water are still contained in the exhaust gas through the desulfurization unit, which may result in the deactivation of the catalyst. Therefore, the effect of SO_2_ and water on the SCR activity of the catalyst was studied. Figure 2 depicts the catalytic performance of Ce_0.35_/TiO_2_ and Ho_0.45_Ce_0.35_/TiO_2_, as a function of time in the presence of 200 ppm SO_2_ and 8 vol.% water at 200°C. As exhibited in figure 2, the NO conversion over Ce_0.35_/TiO_2_ decreased from 52% to 33% after introducing SO_2_ + H_2_O for 200 min, then gradually recovered (37%) after the cut off of SO_2_ + H_2_O and kept stable during the following test period. By contrast, the presence of SO_2_ + H_2_O in the feed gas induced a dramatic decrease of NO conversion over Ho_x_Ce_0.35_/TiO_2_ by 10%. After eliminating SO_2_ + H_2_O from the feed gas, the conversion of NO over Ho_x_Ce_0.35_/TiO_2_ was gradually restored to a certain extent but is less than the initial value (about 72%). All these analyses implied that a better resistance of SO_2_ + H_2_O could be achieved by Ho modification.

### 3.3. Brunauer–Emmett–Teller results

BET surface area, total pore volume and average pore were tested. As listed in table 1, the specific surface areas of Ce_0.35_/TiO_2_, Ho_0.35_/TiO_2_, Ho_0.15_Ce_0.35_/TiO_2_, Ho_0.3_Ce_0.35_/TiO_2_, Ho_0.45_Ce_0.35_/TiO_2_, and Ho_0.6_Ce_0.35_/TiO_2_ are 189.61, 157.34, 196.33, 198.34, 204.56 and 203.65 m^2^ g^−1^, respectively. It is obvious that the specific surface area of Ho_x_Ce_0.35_/TiO_2_ became larger as the Ho/Ti molar ratio increased from 0.15 to 0.45. However, doping excess Ho species to Ce/TiO_2_ (Ho/Ti molar ratio = 0.6) may result in a decrease in BET surface area. Considering the SCR activity results from figure 1*a*, Ce/TiO_2_ with proper Ho species modification may possess higher active surface area, which is beneficial for the effective contacts with reactants.

**Table 1.** Textural parameters of the catalysts.

samples	BET surface area (m^2^ g^−1^)	pore volume (cm^3^ g^−1^)	average pore diameter (nm)
Ce_0.35_/TiO_2_	189.61	0.612	9.55
Ho_0.35_/TiO_2_	157.34	0.424	7.89
Ho_0.15_Ce_0.35_/TiO_2_	196.33	0.608	9.43
Ho_0.3_Ce_0.35_/TiO_2_	198.34	0.627	9.57
Ho_0.45_Ce_0.35_/TiO_2_	204.56	0.628	9.61
Ho_0.6_Ce_0.35_/TiO_2_	203.65	0.611	9.47

### 3.4. Powder X-ray diffraction results

**Figure 3.**
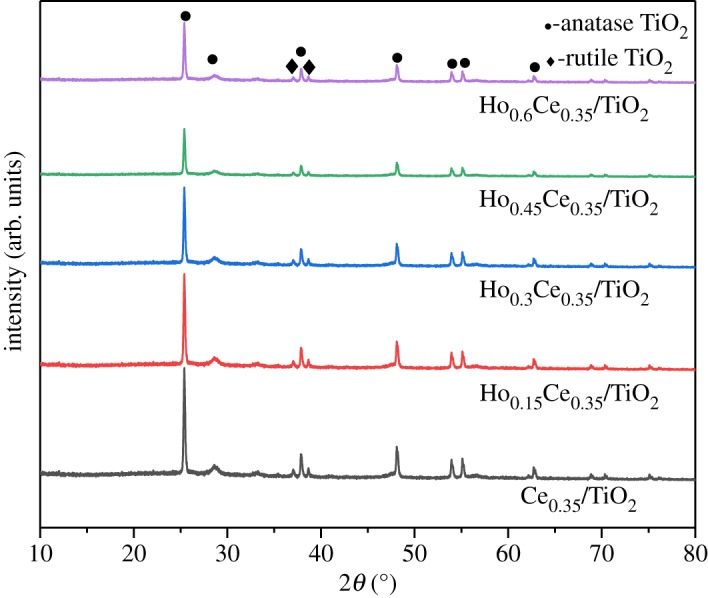
XRD patterns of Ho_x_Ce_0.35_/TiO_2_ catalysts.

XRD patterns of Ce_0.35_/TiO_2_ and Ho_x_Ce_0.35_/TiO_2_ are shown in figure 3. Only diffraction peaks assigned to TiO_2_ are detected. Specifically, much anatase-phase TiO_2_ (PDF-ICDD21-1272) and a little rutile-phase TiO_2_ (PDF-ICDD21-1276) are observed. A similar phenomenon was also reported by Liu *et al*. [21]. It means that Ce and Ho species are highly dispreading on the surface of TiO_2._ With the increase of Ho-doping amount, the intensities of all diffraction peaks became weak, suggesting that the introduction of Ho could further reduce the crystallization of TiO_2_. All of the factors above are favourable to a good SCR performance.

### 3.5. X-ray photoelectron spectroscopy results

**Figure 4.**
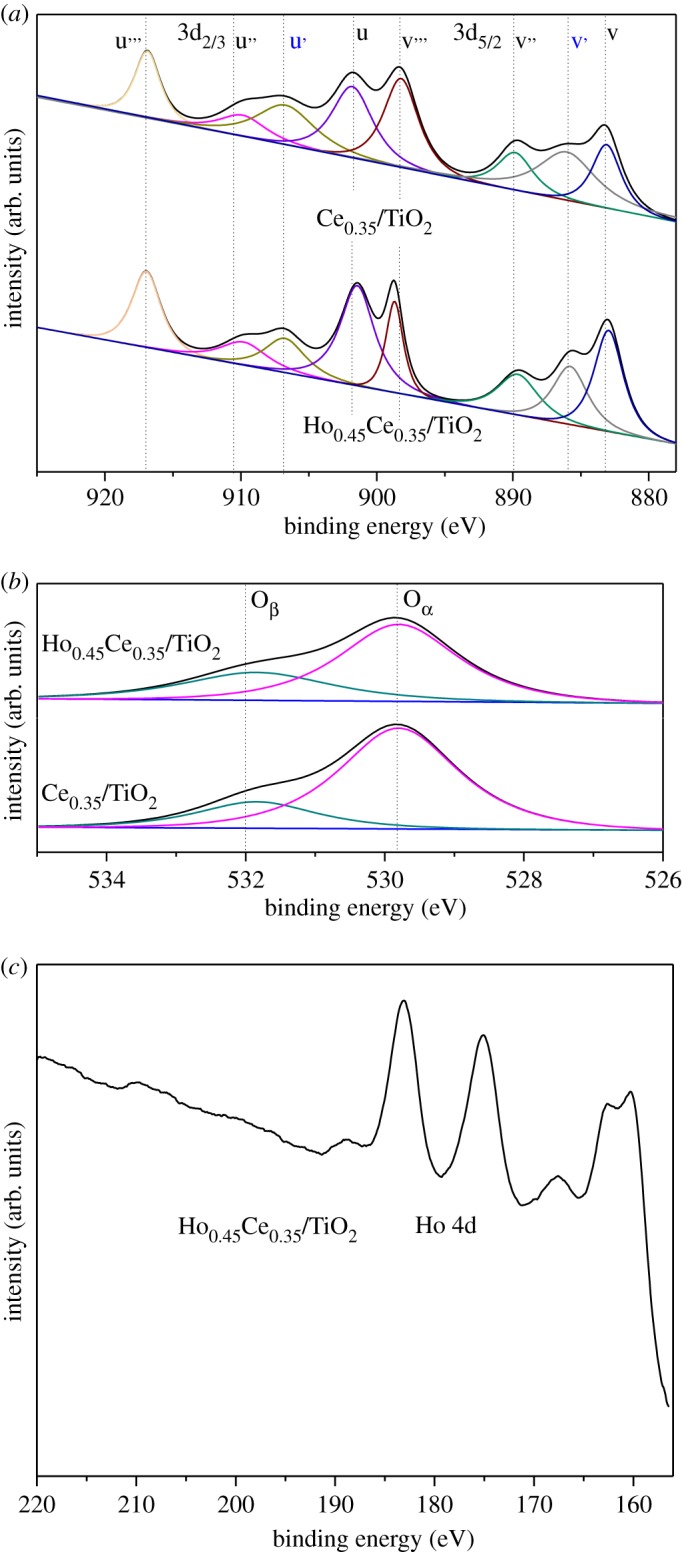
XPS spectra of Ce_0.35_/TiO_2_ and Ho_x_Ce_0.35_/TiO_2_ catalysts.

Figure 4 exhibits the X-ray photoelectron spectroscopy (XPS) spectra of Ce 3d and O 1s over Ce_0.35_/TiO_2_ and Ho_0.45_Ce_0.35_/TiO_2_ catalysts. In addition, the XPS spectrum of Ho 4d over Ho_0.45_Ce_0.35_/TiO_2_ has been given in figure 4*c*. Table 2 lists the surface element compositions and their chemical states by the XPS technique.

**Table 2.** Surface elemental analysis by XPS.

samples	atomic concentration (%)				Ce^3+^/(Ce^3+^ + Ce^4+^)	O*_β_*/(O*_β_* + O*_α_*)
	Ce	Ti	O	Ho		
Ce_0.35_/TiO_2_	4.63	13.22	82.15	—	22.33	23.42
Ho_0.45_Ce_0.35_/TiO_2_	4.56	12.98	80.35	2.11	31.45	33.25

As seen in figure 4*a*, the complicated Ce 3d XPS curves of different samples were made up of eight peaks. u and v peaks belonged to 3d_3/2_ and 3d_5/2_ spin–orbit components, respectively. u’ and v’ peaks could be attributed to Ce^3+^ and the other peaks could be assigned to Ce^4+^ [22]. These Ce^3+^ /Ce^4+^ pairs over the catalyst surface were beneficial for not only the storage and release of active oxygen species but also the oxidation of NO to NO_2_ [23]. Additionally, more Ce^3+^ would promote the generation of more oxygen vacancies, which help to adsorb reactants [24,25]. The factors mentioned above proved to contribute to the progress of the SCR reaction. Thus, it is necessary to study the ratio of Ce^3+^/(Ce^3+^ + Ce^4+^) over the selected catalysts. The ratio of Ce^3+^/(Ce^3+^ + Ce^4+^) was calculated according to the peak area ratio of the Ce^3+^ and Ce^4+^ peaks. The corresponding results are listed in table 2: Ho_0.45_Ce_0.35_/TiO_2_ (31.45%) and Ce_0.35_/TiO_2_ (22.33%). Thus, Ho-doping could promote the transformation of Ce^4+^ to Ce^3+^ over the catalyst surface, which could also effectively improve the SCR activity of Ce_0.35_/TiO_2_.

Figure 4*b* shows that the O 1s XPS spectra of Ce_0.35_/TiO_2_ and Ho_0.45_Ce_0.35_/TiO_2_ consisted of two peaks, lattice oxygen (binding energy = 529.8 eV, labelled as O*_α_*) and chemisorbed oxygen (binding energy = 532 eV, labelled as O*_β_*) [26,27]. It is well recognized that O*_β_* is more active than O*_α_* in the oxidation reactions of NO to NO_2_ [28], which is beneficial for the ‘fast SCR’ reaction (NO + NO_2_ + 2NH_3_ = 2N_2_ + 3H_2_O). ‘Fast SCR’ reaction has been proved conducive to the improvement of the low-temperature SCR activity [29]. The O*_β_*/(O*_α_* + O*_β_*) ratio was calculated and is presented in table 2. It could be observed that Ho_0.45_Ce_0.35_/TiO_2_ has a bigger O*_β_*/(O*_α_* + O*_β_*) ratio than Ce_0.35_/TiO_2_, which meant that chemisorbed oxygen over the catalyst surface of Ce_0.35_/TiO_2_ with Ho modification was obviously improved. Considering the results of the SCR activity and Ce 3d XPS, the O*_β_* ratio result is corresponding with the Ce^3+^ ratio and SCR activity. It may be concluded that more Ce^3+^ was accompanied by an increment of oxygen vacancies and active oxygen species, which played a positive role in the SCR activity. Finally, the XPS spectrum of Ho 4d over Ho_0.45_Ce_0.35_/TiO_2_ is exhibited in figure 4*c*.

### 3.6. H_2_temperature-programmed reduction results

**Figure 5.**
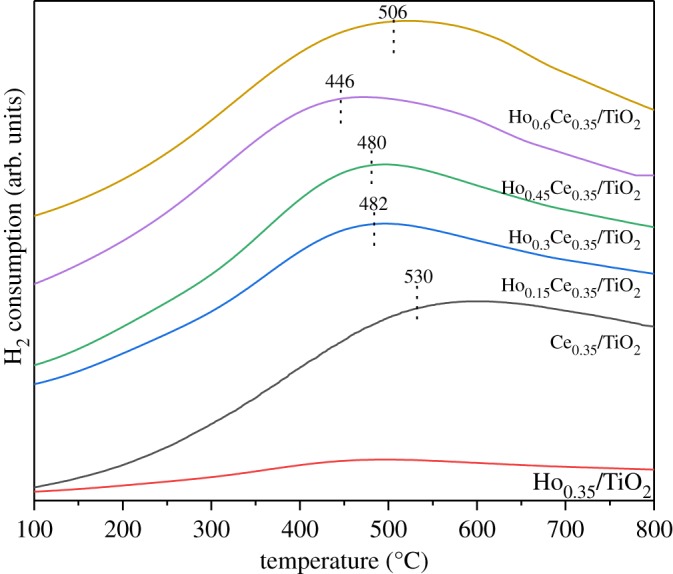
H_2_-TPR patterns of Ho_x_Ce_0.35_/TiO_2_ catalysts.

H_2_-TPR was performed for studying the redox ability of catalysts. In figure 5, no obvious reduction peak of Ho_0.35_/TiO_2_ is observed. The reduction peak of Ce_0.35_/TiO_2_ at about 530°C belonged to the reduction of Ce^4+^ to Ce^3+^ [30,31]. With the introduction of Ho to Ce_0.35_/TiO_2_, the reduction peak of surface Ce^4+^ moved to lower temperature, which could significantly improve the mobility of surface O owing to the strong synergetic effect between Ti, Ce and Ho species. It was also reported that the synergetic effect could lead to the rise of abundant O defects [32,33]. More O defects were beneficial for the improvement of SCR activity because they could promote O diffusion from the subsurface layer and progressively proceed more in-depth into the bulk [34,35]. It could also be observed that Ho_0.45_Ce_0.35_/TiO_2_ showed the lowest reduction temperature at 446°C and this result is corresponding with its best SCR performance. It seems that further increasing the Ho amount would increase the catalyst reduction temperature. In conclusion, the stronger oxidation–reduction ability of Ho_0.45_Ce_0.35_/TiO_2_ is beneficial for the outstanding SCR reaction performance.

### 3.7. NH_3_ temperature-programmed desorption

**Figure 6.**
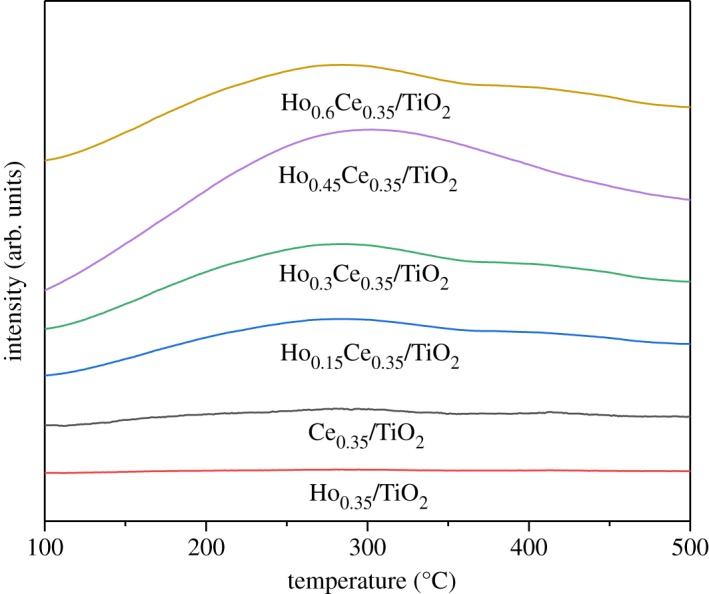
NH_3_-TPD patterns of Ho_x_Ce_0.35_/TiO_2_ catalysts.

Figure 6 shows the effect of Ho modification on NH_3_ desorption behaviour of the prepared samples. From figure 6, no obvious desorption peak of Ho_0.35_/TiO_2_ was observed and the peak area of Ce_0.35_/TiO_2_ is shallow. After the introduction of Ho, the peak surface area gradually increases and the NH_3_-TPD profiles existed as a broad peak with the full range of 120–450°C, which included physically adsorbed NH_3_, the chemically adsorbed species including adsorbed NH_3_ species on Brønsted acid sites and strongly adsorbed on Lewis acid sites [33,36,37]. Thus, more surface sites were available on the Ce_0.35_/TiO_2_ surface for NH_3_ adsorption after introducing Ho, which could be evidenced by the largest desorption peak area of Ho_0.45_Ce_0.35_/TiO_2_. The phenomenon could also indicate that Ho_0.45_Ce_0.35_/TiO_2_ possesses the most potent surface acidity. Thus the adsorption of NH_3_ over it could be boosted and the SCR activity could be promoted correspondingly.

### 3.8. NO oxidation

**Figure 7.**
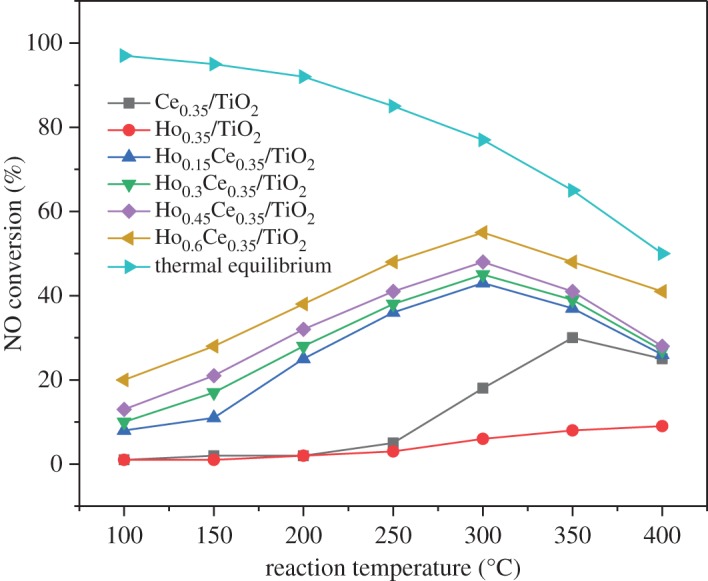
Oxidation activity of NO to NO_2_ by O_2_ over different catalysts (500 ppm NO, 3 vol.% O_2_ and 200 ml min^−1^ total flow rate).

Figure 7 exhibits the NO conversion of NO oxidation reaction over the prepared catalysts. It could be easily seen that the NO oxidation conversions over Ce_0.35_/TiO_2_ and Ho_0.35_/TiO_2_ are very low (below 25%) during 100–400°C, which is consistent with the lowest SCR activity due to the inefficient conversion from NO to NO_2_. The activity curves of other catalyst samples demonstrate a parabolic trend, which is an indication of the conversion from the kinetically controlled regime to thermo-dynamically controlled regime [38]. Especially, Ho_0.45_Ce_0.35_/TiO_2_ has a more significant effect on NO oxidation than other samples. The formation of more NO_2_ on the catalyst surface facilitates NOx reduction in the low-temperature range, which was also corresponding with the XPS results. Although Ho_0.6_Ce_0.35_/TiO_2_ had the highest oxidation activity of NO to NO_2_ of all samples, it exhibited a relatively lower de-NOx activity compared with Ho_0.45_Ce_0.35_/TiO_2_, which may be attributed to its decreased specific surface area leading to the decreased adsorbed NH_3_ species.

### 3.9. *In situ* diffuse reflectance infrared Fourier transform spectroscopy results

#### 3.9.1. NH_3_ adsorption

**Figure 8.**
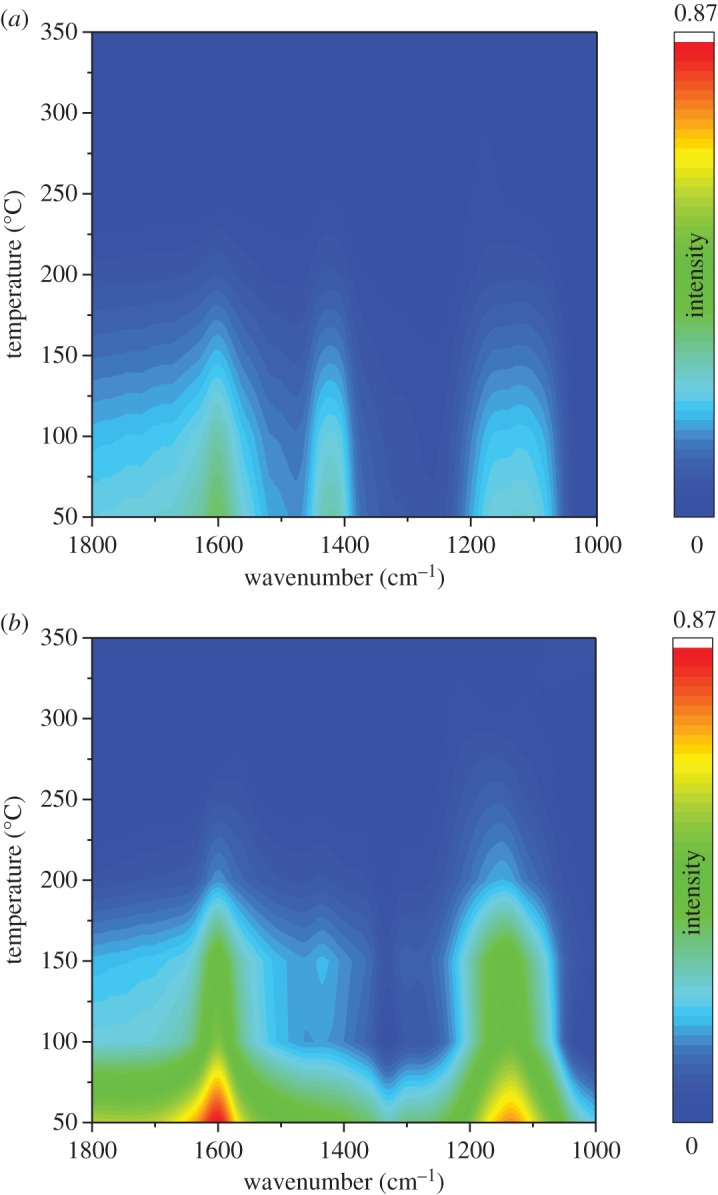
*In situ* DRIFTS of NH_3_ adsorption with increasing temperature from 50 to 350°C: (*a*) Ce_0.35_/TiO_2_ and (*b*) Ho_0.45_Ce_0.35_/TiO_2_.

Figure 8*a* shows the DRIFT spectra of NH_3_ adsorption over Ce_0.35_/TiO_2_ at different temperatures. The bands at 1599, 1161 cm^−1^ with a shoulder at 1109 cm^−1^ attributed to the coordinated NH_3_ linked to Lewis acid sites (NH_3_-L) [39,40] could be observed. The band at 1418 cm^−1^ could be assigned to NH_4_^+^ species on Brønsted acid sites (NH_4_^+^-B). Notably, all the bands linked to NH_3_ species decrease with the temperature increasing owing to the desorption effect.

Figure 8*b* exhibits the DRIFT spectra of NH_3_ adsorption over Ho_x_Ce_0.35_/TiO_2_. Similar to the spectra over Ho_x_Ce_0.35_/TiO_2,_ the NH_3_-L bands (1599 and 1143 cm^−1^) and the NH_4_^+^-B band (1432 cm^−1^) could also be seen. However, the band intensity of adsorbed NH_3_ over Ho_x_Ce_0.35_/TiO_2_ was much stronger than that over Ce_0.35_/TiO_2,_ which indicated that the introduction of Ho species could greatly increase the quantity of both Lewis acid sites and Brønsted acid sites. Previous study by Chen *et al*. [41] and Zhou *et al*. [42] reported that more Brønsted acid sites could help in the generation of adsorbed NH_3_ species, thus promoting the low-temperature SCR performance. It should also be noted that the intensity of the bands at 1432 cm^−1^ assigned to Brønsted acid sites in figure 8*b* decreases faster with temperature rising in comparison with those assigned to Lewis acid sites, suggesting NH_3_ bonded to Lewis acid sites possessed a better thermostability than that bonded to Brønsted acid sites [41].

#### 3.9.2. NO + O_2_ adsorption

**Figure 9.**
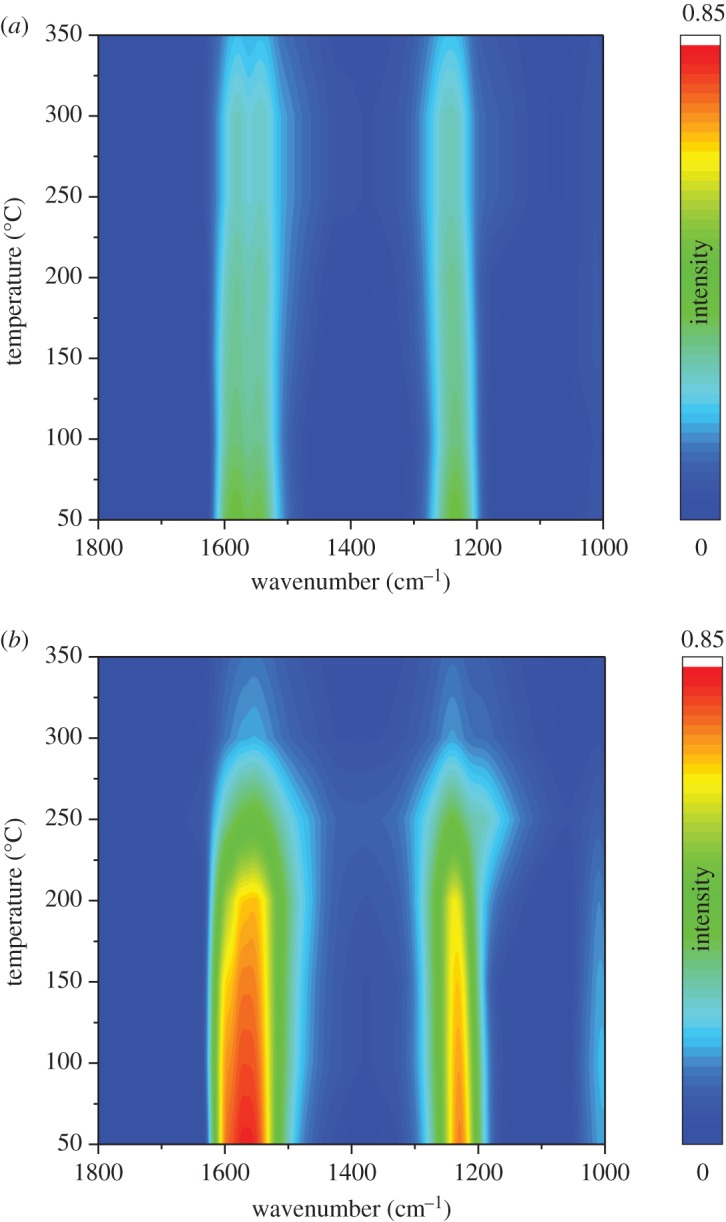
*In situ* DRIFTS of NO + O_2_ adsorption with increasing temperature from 50 to 350°C: (*a*) Ce_0.35_/TiO_2_ and (*b*) Ho_0.45_Ce_0.35_/TiO_2_.

Figure 9*a* shows the DRIFT spectra of NO + O_2_ adsorption over Ce_0.35_/TiO_2_ at different temperatures. The bands at 1577, 1536 cm^−1^ attributed to bidentate nitrate could be clearly observed; the band at 1599 cm^−1^ could be assigned to ad-NO_2_ and the band at 1241 cm^−1^ could be assigned to bridging nitrates [43–45]. It could be observed that all the bands decrease with the temperature increasing owing to the drop in thermal stability.

Figure 9*b* exhibits the DRIFT spectra of NO + O_2_ adsorption over Ho_0.45_Ce_0.35_/TiO_2_ at different temperatures. As shown in figure 9*b*, the peaks at 1600 cm^−1^ and 1564 cm^−1^ belonged to ad-NO_2_ and bidentate nitrate. The peak at 1232 cm^−1^ belonged to bridging nitrates [44]. In comparison with that shown in figure 9*a*, the peak intensity of Ho_0.45_Ce_0.35_/TiO_2_ was stronger than that of Ce_0.35_/TiO_2_, which meant that Ho-doping could greatly improve NOx adsorption of Ce_0.35_/TiO_2_ catalyst.

### 3.10. Promotion mechanism

As evidenced by electronic supplementary material, figure S1, all the adsorbed reactants, including ad-NH_3_ and ad-NO_X_ on Ho_0.45_Ce_0.35_/TiO_2_, could participate in the NH_3_-SCR reaction. Considering all analysis results given above, doping proper amount of Ho into Ce_0.35_/TiO_2_ could generate more active NH_3_ and NO_x_ species on its surface. After adding Ho species, the generation of more Ce^3+^ and O*_β_* over Ho_0.45_Ce_0.35_/TiO_2_ has a facilitation effect on the conversion from NO to NO_2_. Thus, the Langmuir–Hinshelwood (L–H) mechanism and Eley–Rideal (E–R) mechanism should be mainly responsible for the promoted low-temperature NH_3_-SCR activity over Ho_0.45_Ce_0.35_/TiO_2_, which could be described by the following processes:

(1) L–H mechanism:
  3.1 $$\mathrm{NO}+O_{2}(g)\to {\mathrm{NO}}_{2}(\mathrm{ad})$$
  3.2 $$NH_{3}(g)\overset{Ce^{4+}}{\to }NH_{3}(\mathrm{ad})\;\;(\mathrm{Lewis}\;\mathrm{acid}\;\mathrm{sites}).$$

‘Fast SCR’ reaction:

3.3 $${\mathrm{NO}}_{2}(\mathrm{ad})+2NH_{3}(\mathrm{ad})+\mathrm{NO}(g)\to 2N_{2}(g)+3H_{2}O(g)$$

3.4 $$NH_{3}(g)\overset{Ce^{3+}}{\to }{\mathrm{NH}}_{4}^{+}\;(\mathrm{ad})\;\text{over Brønsted acid sites}$$

3.5 $$\;{\mathrm{NH}}_{4}^{+}+e^{-}+{\mathrm{NO}}_{2}(\mathrm{ad})\to NH_{4}{\mathrm{NO}}_{2}(\mathrm{ad})\to N_{2}+H_{2}O.$$

(2) E–R mechanism:
  3.6 $$NH_{3}(g)\overset{Ce^{4+}}{\to }NH_{3}(\mathrm{ad})\;(\mathrm{on}\;\mathrm{Lewis}\;\mathrm{acid}\;\mathrm{sites})$$
  3.7 $$O_{2}(g)\to 2O(\mathrm{ad})$$
  3.8 $$NH_{3}(\mathrm{ad})+O(\mathrm{ad})\to NH_{2}(\mathrm{ad})+\mathrm{OH}(\mathrm{ad})$$
  3.9 $$\mathrm{NO}(g)+NH_{2}(\mathrm{ad})\to NH_{2}\mathrm{NO}(\mathrm{ad})$$
  3.10 $$NH_{2}\mathrm{NO}(\mathrm{ad})\to N_{2}(g)+H_{2}O.$$

## 4. Conclusion

In summary, Ce_0.35_/TiO_2_ modified with a certain amount of Ho shows an outstanding low-temperature SCR performance and superior SO_2_ + H_2_O durability, which could boost the practical application of Ce/TiO_2_. *In situ* DRIFTS results revealed that the introduction of Ho species could efficiently promote both active ad-NH_3_ and ad-NOx species on Ce_0.35_/TiO_2._ Moreover, all of these could contribute to the low-temperature SCR activity of Ce_0.35_Ho_0.45_/TiO_2_ through L–H route and E–R route.

## Supplementary Material

In situ DRIFTS of NO + O2 reacted with pre-adsorbed NH3 species (A) and NH3 reacted with pre-adsorbed NOx species (B) at 200 °C on the Ho0.45Ce0.35/TiO2 catalyst.

Reviewer comments

## Data accessibility

Our data are deposited at: http://dx.doi.org/10.5061/dryad.c86d5m0 [46].
