# Supplementary material for: Enhanced low-temperature NH3-SCR performance of Ce/TiO2 modified by Ho catalyst
Source: R Soc Open Sci. 2019 Mar 13;6(3):182120. doi: 10.1098/rsos.182120 (PMC6458355; doi:10.1098/rsos.182120)
Supplement: In situ DRIFTS of NO + O2 reacted with pre-adsorbed NH3 species (A) and NH3 reacted with pre-adsorbed NOx species (B) at 200 °C on the Ho0.45Ce0.35/TiO2 catalyst. [file rsos182120supp1.docx]

Supporting Information

Fig S1. *In situ* DRIFTS of NO + O_2_ reacted with pre-adsorbed NH_3_ species (A) and NH_3_ reacted with pre-adsorbed NOx species (B) at 200 °C on the Ho_0.45_Ce_0.35_/TiO_2_ catalyst.

Fig S1. *In situ* DRIFTS of NO + O_2_ reacted with pre-adsorbed NH_3_ species (A) and NH_3_ reacted with pre-adsorbed NOx species (B) at 200 °C on the Ho_0.45_Ce_0.35_/TiO_2_ catalyst. (500 ppm NO, 500 ppm NH_3_, 3 vol.% O_2_, N_2_ balance)
